# Resting state functional connectivity provides mechanistic predictions of future changes in sedentary behavior

**DOI:** 10.1038/s41598-021-04738-y

**Published:** 2022-01-18

**Authors:** Timothy P. Morris, Aaron Kucyi, Sheeba Arnold Anteraper, Maiya Rachel Geddes, Alfonso Nieto-Castañon, Agnieszka Burzynska, Neha P. Gothe, Jason Fanning, Elizabeth A. Salerno, Susan Whitfield-Gabrieli, Charles H. Hillman, Edward McAuley, Arthur F. Kramer

**Affiliations:** 1 Department of Psychology, Northeastern University, 435 ISEC, 360 Huntington Avenue, Boston, 02115 USA; 2 Department of Neurology and Neurosurgery, Montreal Neurological Institute, McGill University, Montreal, Canada; 3 Brigham and Women’s Hospital, Harvard Medical School, Cambridge, USA; 4 Department of Human Development and Family Studies, Colorado State University, Fort Collins, USA; 5 Beckman Institute for Advanced Science and Technology, University of Illinois at Urbana Champaign, Urbana, USA; 6 Department of Kinesiology and Community Health, University of Illinois at Urbana-Champaign, Urbana, IL USA; 7 Department of Health and Exercise Sciences, Wake Forrest University, Winston-Salem, NC USA; 8 Division of Public Health Sciences, Department of Surgery, Washington University School of Medicine in St. Louis, St. Louis, MO USA; 9 McGovern Institute for Brain Research, Department of Brain and Cognitive Sciences, Massachusetts Institute of Technology, Cambridge, MA USA; 10 Department of Physical Therapy, Movement, and Rehabilitation Sciences, Northeastern University, Boston, MA USA

**Keywords:** Computational neuroscience, Cognitive neuroscience, Attention, Cognitive control, Decision, Human behaviour

## Abstract

Sedentary behaviors are increasing at the cost of millions of dollars spent in health care and productivity losses due to physical inactivity-related deaths worldwide. Understanding the mechanistic predictors of sedentary behaviors will improve future intervention development and precision medicine approaches. It has been posited that humans have an innate attraction towards effort minimization and that inhibitory control is required to overcome this prepotent disposition. Consequently, we hypothesized that individual differences in the functional connectivity of brain regions implicated in inhibitory control and physical effort decision making at the beginning of an exercise intervention in older adults would predict the change in time spent sedentary over the course of that intervention. In 143 healthy, low-active older adults participating in a 6-month aerobic exercise intervention (with three conditions: walking, dance, stretching), we aimed to use baseline neuroimaging (resting state functional connectivity of two a priori defined seed regions), and baseline accelerometer measures of time spent sedentary to predict future pre-post changes in objectively measured time spent sedentary in daily life over the 6-month intervention. Our results demonstrated that functional connectivity between (1) the anterior cingulate cortex and the supplementary motor area and (2) the right anterior insula and the left temporoparietal/temporooccipital junction, predicted changes in time spent sedentary in the walking group. Functional connectivity of these brain regions did not predict changes in time spent sedentary in the dance nor stretch and tone conditions, but baseline time spent sedentary was predictive in these conditions. Our results add important knowledge toward understanding mechanistic associations underlying complex out-of-session sedentary behaviors within a walking intervention setting in older adults.

## Introduction

In 2007 it was estimated that ~ 5.3 million global deaths from non-communicable diseases could have been prevented if people engaged in sufficient levels of moderate-to-vigorous physical activity instead of being insufficiently active^1^. Compounding this further, global statistics show the prevalence of physical inactivity is increasing^2,3^. Over a third of the US population (34.8%) lead sedentary lifestyles^2–4^ and the economic burden caused by physical inactivity is estimated to cost private and public health-care systems $53.8 billion per year^5,6^.

To combat the negative consequences of sedentary behaviors, particularly in older adults, the field has studied extensively the beneficial effects of exercise interventions^7–9^. The most well studied exercise interventions are walking interventions, which are both economical and easily accessible, particularly for older adults^10^. These studies have led to numerous discoveries on the beneficial effects of increased walking on cognitive function, particularly, processing speed, memory and executive function^11^. Walking interventions also have been shown to increase hippocampal volume^12^ and the plasticity of functional brain networks^13^. These results are particularly important given that these same outcomes are also associated with age-related decline^14–17^. However, engaging in a significant behavioral change is non-trivial and despite significant efforts to understand determinants of sedentary lifestyles, the prevalence of physical inactivity continues to increase^2,3^.

Sedentary behaviors are not simply the inverse of moderate-to-vigorous physical activity^18,19^. For example, a person can both perform 30 min of moderate-to-vigorous physical activity achieving recommended levels^20^ and also engage in a high volume of sedentary behavior throughout the rest of the day. Further, the determinants of sedentary behaviors are distinct from those of physical activity engagement too^18^. For example, to engage in a bout of physical activity one must inhibit a desire to minimize effort one time, whereas avoiding sedentary behaviors throughout the day requires consistent awareness and self-regulation of such behaviors^21^. Understanding the determinants of sedentary behaviors has relied upon psychological frameworks and cognitive-behavioral theories^18,21–23^. Automatic processes, attitudes and habits and self-regulation have been suggested to regulate daily sedentary behavior^21^. Associations between self-efficacy and sedentary behavior has been shown in meta-analyses^24^, and interventions targeting perceptions of competence and capability (self-efficacy) have been shown to reduce time spent sedentary in younger adults^25^. From a cognitive perspective, to successfully overcome short-term costs in favor of longer-term benefits (like reducing sedentary behaviors), executive control functions, such as inhibitory control, flexibility and goal-orientated decision making are required^26^. Notwithstanding, multicomponent approaches to intervention development have been conceived^27^ based on this prior knowledge, yet efficacious interventions that lead to sustained behavioral change are yet to be developed. Little emphasis has been placed on the discovery of mechanistic determinants of sedentary behaviors that would provide tangible targets for intervention development and testing.

Behavioral choices involving the assessment of motor costs are ever present in day-to-day life and involve the integration of information about available energy resources to weigh physical and motor costs against expected rewards^28^. A theory of energetic cost minimization postulates that we have an innate attraction towards effort minimization whilst maximizing reward^28–31^. This theory is reflected in evolutionary, developmental and situational scenarios, where for example, humans have developed body shapes and neural circuitry refined for energy optimization^32^, and during development, energy efficient movements are consolidated through motor practice^33^, which are constantly adapted in real time to minimize energy costs, such as gait refinements during walking^34^. Neural circuitry underlying the valuation of potential behaviors related to physical effort costs have consistently implicated both the anterior mid-cingulate cortex (aMCC^35^) and the dorsal anterior insula (dAI) in these behaviors^28,31,36^. For example, in rodents, local field potentials in and coherence between the aMCC and the dAI correlate with relative performance on a physical effort-based task^36^. In humans, neuroimaging studies have demonstrated that the aMCC is a critical region for decision-making of choices involving motor-costs^28^ and further, that activity in the aMCC and the dAI represent the devaluation of rewards associated with physical effort^31^. Additionally, these same regions are consistently implicated in inhibitory control^37,38^, a higher order executive function shown to be needed to overcome physical effort minimization^26^. Together, this theoretical and experimental evidence may suggest a role for the aMCC and the dAI in the regulation of sedentary behaviors.

The discovery of neural predictors of future sedentary behaviors may provide both strong predictive strength as well as mechanistic information relevant for intervention development. The utility and efficacy of functional connectivity (FC) to predict future behavioral outcomes has been demonstrated in previous research. For example, Saghayi and colleagues predicted adherence to mental training programs using FC^39^ and Whitfield-Gabrieli and colleagues predicted treatment response in social anxiety disorder with FC, better than clinical measures alone^40^.

The aim of this present study therefore was to evaluate if the FC of two a-priori defined brain regions (aMCC and the r-dAI) implicated in inhibitory control and physical effort decision making, at baseline, could predict future change in objectively measured sedentary behavior in older adults participating in a 6-month randomized controlled trial of exercise (which included a walking, a dancing and a stretching control condition).

## Methods

### Participants and study design

This study presents results of a secondary analysis of baseline data from participants who participated in a 6-month randomized controlled exercise trial (clinical study identifier: NCT01472744, November 16, 2011). The study procedures were approved by the University of Illinois Institutional Review Board and written informed consent was obtained from all participants prior to any research activities. All methods were carried out in accordance with the Declaration of Helsinki. Healthy but low active older adults were recruited in Champaign County. Two hundred and forty-seven (169 women) low-active (less than two bouts of self-reported moderate exercise per week within the past 6 months) older adults met inclusion criteria for the initial clinical trial. Of which one hundred and sixty-five underwent functional magnetic resonance imaging (fMRI). Participants in the initial trail were randomized to one of four intervention groups; a walking intervention, a walking intervention plus a dietary supplement, a dancing intervention and a control stretch and toning intervention. For the purpose of this analysis, we combined the two walking groups to increase the sample size as the walking portion of the intervention as identical and no significant differences in outcome measures or demographics was found between these two groups (supplementary material 1). All groups met for approximately one hour three times per week for six months. For this analysis, we excluded participants who did not adhere to more than 50% of the intervention sessions (n = 9), for having incomplete accelerometer data available (n = 7), high motion artefact in the fMRI scan (see below for criteria, n = 2), or for influential outlier data points in the outcome variable (see criteria below, n = 4). 143 participants were ultimately included in this study. For more details on this clinical trial, its primary outcomes and neuroimaging data, please refer to earlier work^41–44^. Initially, to enroll in the study, participants must have met the following criteria: were between the ages of 60 and 80 years old, free from psychiatric and neurological illness and had no history of stroke, transient ischemic attack, or head trauma, scored < 23 on the Mini-Mental State Exam, < 21 on a Telephone Interview of Cognitive Status questionnaire and < 10 on the Geriatric Depression Scale, at least 75% right-handed based on the Edinburgh Handedness Questionnaire (a criterion related to functional magnetic resonance imaging (MRI) analyses), demonstrated normal or corrected-to-normal vision of at least 20/40 and no color blindness, screened for safe participation in an MRI environment (e.g., no metallic implants that could interfere with the magnetic field or cause injury and no claustrophobia) and reported to have participated in no more than two bouts of moderate exercise per week within the past 6 months (with the goal of recruiting low active older adults). Our current analysis asks a novel question of this dataset that has not been previously assessed. Table 1 contains complete characterization of the study participants broken down by each intervention group.

**Table 1:** Participant characteristics.

	Walk	Dance	Stretch and Tone	*P*
N	63	40	40	
Age (mean (SD))	65.33 (4.53)	66.15 (4.74)	65.72 (4.89)	0.683
Baseline sedentary time (mean (SD))	537.43 (91.67)	530.11 (92.84)	564.79 (75.35)	0.170
Post sedentary time (mean (SD))	555.72 (107.9)	547.24 (83.03)	574.90 (72.30)	0.388
Female sex (%)	46 (71.9)	28 (70.0)	28 (70.0)	0.970
Increase in sedentary time (%)	38 (60.3)	24 (60.0)	22 (55.0)	0.851

Baseline sedentary time = estimated baseline average daily minutes spent sedentary, Post sedentary time = estimated post-intervention average daily minutes spent sedentary. P-value represents the results of ANOVA (continuous) or chi-square test of independence (categorical) tests on outcome and demographic variables between groups.

### Accelerometry

Time spent sedentary was measured using an ActiGraph accelerometer device (Model GT1M or GT3X; ActiGraph, Pensacola, FL) for one week at baseline and one-week post-intervention. Participants were instructed to wear the accelerometer on the nondominant hip during waking hours for seven consecutive days. For data reduction, the following criteria were applied to the raw data recorded by each monitor: wear time validation criterion of ≥ 10 h of wear time per day for at least 3 days and an interruption period of 60 min^45^. These data were downloaded as activity counts, which represent raw accelerations summed over a specific epoch length (e.g., 1 s) and subsequently processed into activity intensities in ActiLife software package (Version 6; Actigraph, Pensacola, FL). A low intensity proxy for sedentary behavior was derived using older adult-specific cut points^46^ such that 50 or fewer counts per minute corresponded with sedentary behavior. Estimated average daily minutes spent in the sedentary category (< 50 counts/min) were calculated by dividing the number of minutes spent in that category by the total number of valid days worn per participant. Our outcome measure (change in time spent sedentary) was calculated as post-test minus pre-test of the estimated average daily minutes spent sedentary.

### Magnetic resonance imaging: preprocessing

Participants underwent an MRI scanning session in a 3 Tesla Siemens TIM Trio system with a 12-channel head coil. High-resolution structural MRI scans were acquired using 3D MPRAGE T1-wighted sequences (TR = 1900 ms; TE = 2.32 ms; TI: 900 ms; flip angle = 9°; matrix = 256 × 256; FOV = 230 mm; 192 slices; resolution = 0.9 × 0.9 × 0.9 mm; GRAPPA acceleration factor 2). One run of T2*-weighted resting state echoplanar imaging (EPI) data was obtained with the following parameters: (6 min, TR = 2 s, TE = 25 ms, flipangle = 80°, 3.4 × 3.4 mm^2^ in-plane resolution, 35 4 mm-thick slices acquired in ascending order, Grappa acceleration factor = 2, 64 × 64 matrix).

Preprocessing of the functional resting state data was performed using the CONN-toolbox v.19c^47^, relying upon SPM v.12 (Wellcome Department of Imaging Neuroscience, UCL, London, UK) in MATLAB R2019a (The MathWorks Inc, Natick, MA, USA). The latest default preprocessing pipeline implemented in Conn was performed which consists of the following steps: functional realignment and unwarping, slice timing correction, outlier identification, segmentation (into grey matter, white matter and cerebrospinal fluid) and normalization into standard Montreal Neurologic Institute (MNI) space resampled to 2 mm isotropic voxels for functional data and 1 mm for anatomical data, using 4th order spline interpolation. Functional scans were spatially smoothed using a 6 mm FWHM Gaussian kernel. During the outlier detection step, acquisitions with framewise displacement above 0.9 mm or global BOLD signal changes above 5 standard deviations were flagged as outliers using the Artefact Detection Tools (www.nitrc.org/projects/artifact_detect). Two participants were removed from the final analyses for having > 30 scan volumes flagged. This cut off was determined based on preserving at least 5 min of scanning time^48^. Additionally, mean motion (framewise displacement) was used as a covariate of no interest in all second level analyses. This was done to be over conservative given previous studies have shown high degree of motion-behavior correlations^49^, despite the fact that no motion parameter was significantly correlated with sedentary time in our data (*P* > 0.05). Denoising of the functional data was performed using a principal component analysis-based correction method, CompCor^50^. Linear regression was used to remove the effects of these artifacts on the BOLD time series for each voxel and each subject taking into account noise components from cerebral white matter and cerebrospinal fluid, estimated subject-motion parameters (3 rotation and 3 translation parameters and 6 other parameters representing their first order time derivatives), scrubbing (one noise component for each outlier scan detected in the outlier detection step) and constant and first-order linear session effects. Temporal band-pass filtering (0.008–0.09 Hz) was applied to remove physiological, subject-motion and outlier-related artefacts. MRI quality control measures are found in the supplementary material 2.

### Seed-based correlations

**Figure 1:**
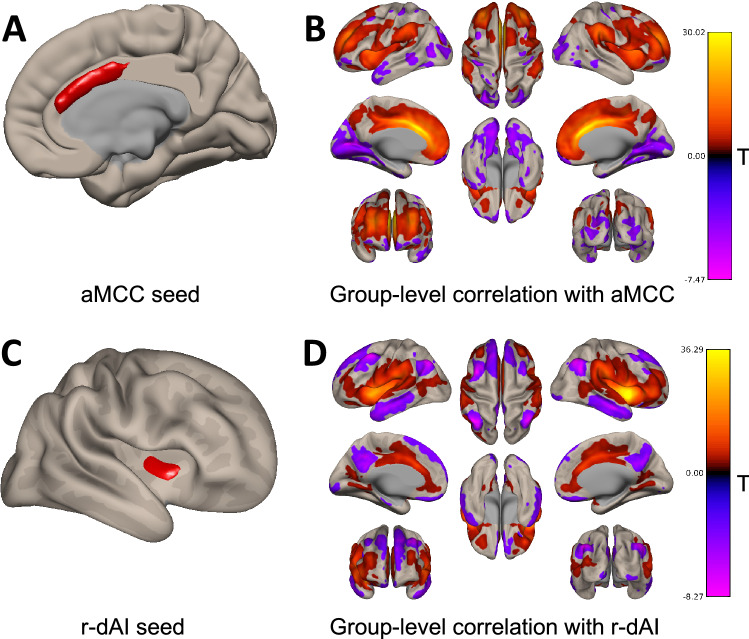
(**A**) Illustrates the aMCC seed region. (**B**) Summary figure of the whole group-level connectivity with the aMCC seed ROI showing functional connectivity with regions of the salience network (e.g. anterior insula, temporoparietal junction). (**C**) Illustrates the r-dAI seed region. (**D**) Summary figure of the whole-group-level connectivity with the r-dAI seed ROI demonstrating our seed functionally connected to the salience network (bilateral insula, temporoparietal junction, inferior frontal operculum, anterior cingulate cortex), and was anticorrelated with the default mode network (inferior parietal lobule, precuneus, superior frontal gyrus). All second-level contrasts assessing the association with behavioral variables of interest take the average BOLD signal within the seed region only and correlate that with all other voxel in the brain mask.

The average time series in two regions of interest (ROI), the anterior mid-cingulate (aMCC) and the right dorsal anterior insula (r-dAI) were extracted. We defined our seeds using the 100-parcel functional atlas by Schaefer 2018. Because the functional parcels of the aMCC and the r-dAI extend outside of the anatomical boundaries of interest, we limited our seed ROIs to just the functional parcel constrained by the anatomical boundaries of the aMCC and the r-dAI set by the Harvard–Oxford anatomical atlas. This was done by binarizing the parcels from each atlas and using ‘fslmaths’ functions (Functional Magnetic Resonance Imaging of the Brain's Software Library, http://www.fmrib.ox.ac.uk/fsl) to multiply the two parcels together (see Fig. 1 for an illustration of the seed ROIs). Then, Pearson’s correlation coefficients were computed between the average time series in each ROI and the time series of all other voxels in the brain and converted to normally distributed z-scores using Fisher transformation prior to performing the second-level general linear model. Individual change in sedentary time was entered as a covariate of interest in the second-level analysis, controlling for nuisance variables, age, gender, baseline sedentary time and mean framewise displacement, in separate general linear models for each ROI. In a confirmatory step, results in this second level analyses were estimated using a height threshold (voxel level *P* < 0.001) and a family-wise corrected cluster-extent threshold (*p* FWE < 0.05) and can be found in the supplementary materials 3.

### Statistical analyses

The effect of each intervention on time spent sedentary was assessed using repeated measures analysis of variance. Differences in outcome and demographic variables between groups were assessed using analysis of variance for continuous outcomes and chi-square test of independence for categorical variables within the Table1 function in R. The Breusch-Pagan Test of Heteroskedasticity was performed to ensure homogeneity of variance.

To assess whether baseline measure of sedentary time predicted change in time spent sedentary we ran independent linear regression models using leave-one-out cross validation (LOOCV) in each group with age and sex, (and baseline sedentary time in the FC models). Model assumptions for linear models were checked using Q-Q and fitted vs. residual plots in R. The significant influence of outliers was checked using Cook’s distance with a cut off of 0.5 (n = 3 for the stretch and tone group and n = 1 for the walking group).

To test whether seed-based functional connectivity predicted change in time spent sedentary we implemented a nested cross validation procedure. Each outer-layer LOO iteration used data from N-1 subjects to: (a) first select the largest cluster of voxels showing significant (*P* < 0.001) voxel-level associations with time spent sedentary; (b) run an inner-layer cross-validation procedure to fit a linear model between average connectivity in that cluster and time spent sedentary; and (c) compute the average connectivity within this cluster for the left-out subject and use the estimated linear model parameters to predict time spent sedentary for this same left-out subject.

Model performance is presented as cross-validated R^2^ values. We also present the average prediction error (RMSE) which represents the difference between the observed and predicted values. Statistical significance of the prediction models was assessed via 1000 nonparametric permutations and the p-value of the permutation tests were calculated as the proportion of sampled permutations that are greater or equal to the true prediction correlation.

LOOCV of the seed-based correlation clusters was performed in MATLAB using the “spm_nestedcrossvalidation” code and all other statistics performed in RStudio Version 3.6.3 (R Foundation for Statistical Computing, Vienna, Austria) using “tidyverse”^51^, “Caret”^52^ and base R packages.

### Ethics approval

The University of Illinois Institutional Review Board approved all procedures used in the study.

### Consent to participate

All participants gave written informed consent before participation in any study procedures, all of which conformed to the Declaration of Helsinki for research involving human subjects.

### Consent for publication and author responsibilities

All authors agree to the contents of this manuscript and give consent for its publication.

## Results

**Figure 2:**
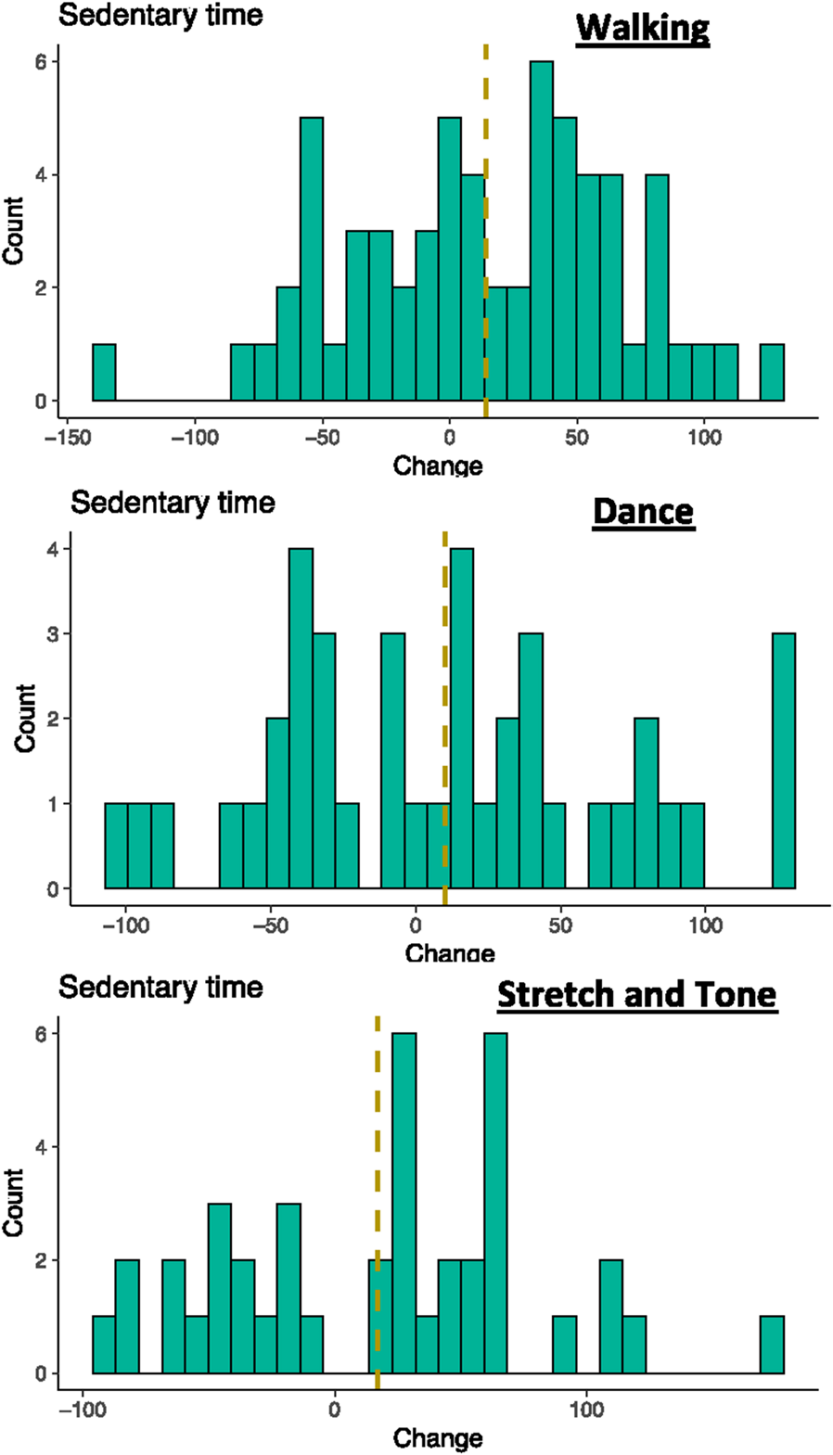
Histograms of participant changes in sedentary time over the 6-month interventions. A numerically similar proportion of individuals increased as decreased their time spent sedentary. Gold vertical line represents the mean change, “0” on the x-axis represents no change.

One-hundred and forty-three low-active healthy older adults were included in this study. Table 1 outlines participant demographics broken down by intervention condition. The distribution of the change in time spent sedentary (Fig. 2) revealed that a higher proportion of participants increased their time spent sedentary over the course of the intervention with no significant differences in this proportion between intervention conditions (Table 1). No main effect of condition assignment (F(_1)_ = 1.981, *P* = 0.167), time (F(_1)_ = 2.934, *P* = 0.087) or time by condition interaction (F(_1)_ = 0.137, *P* = 0.711) was found for time spent sedentary over the course of the intervention.

### Baseline time spent sedentary

Baseline time spent sedentary predicted change in time spent sedentary in the stretch and tone and dance groups, but not the walking group (Table 2).

**Table 2:** Prediction of change in time sent sedentary.

	*β*	*SE*	P	*R*^2^	*RMSE*
**Walking group**					
Baseline sed time	− 0.099	0.079	0.22	0.06	56.36
aMCC FC	213.7	45.88	.002	0.11	59.13
r-dAI FC	173.01	33.70	.021	0.11	45.73
**Dance group**					
Baseline sed time	− 0.36	0.115	0.003*	0.10	57.56
aMCC FC	n/a	n/a	n/a	n/a	n/a
r-dAI FC	n/a	n/a	n/a	n/a	n/a
**Stretch and tone group**					
Baseline sed time	− 0.370	0.094	< 0.001*	0.20	55.09
aMCC FC	n/a	n/a	n/a	n/a	n/a
r-dAI FC	n/a	n/a	n/a	n/a	n/a

All models are performed using leave-one-out cross validation. *RMSE* root mean square error and represents the differences between the observed and predicted outcomes (the lower the value the better the prediction). All significant models survive multiple comparisons using false discovery rate (supplementary material 4). Statistical significance of the prediction models was assessed via 1000 nonparametric permutations and the p-value of the permutation tests were calculated as the proportion of sampled permutations that are greater or equal to the true prediction correlation.

### Functional connectivity

**Figure 3:**
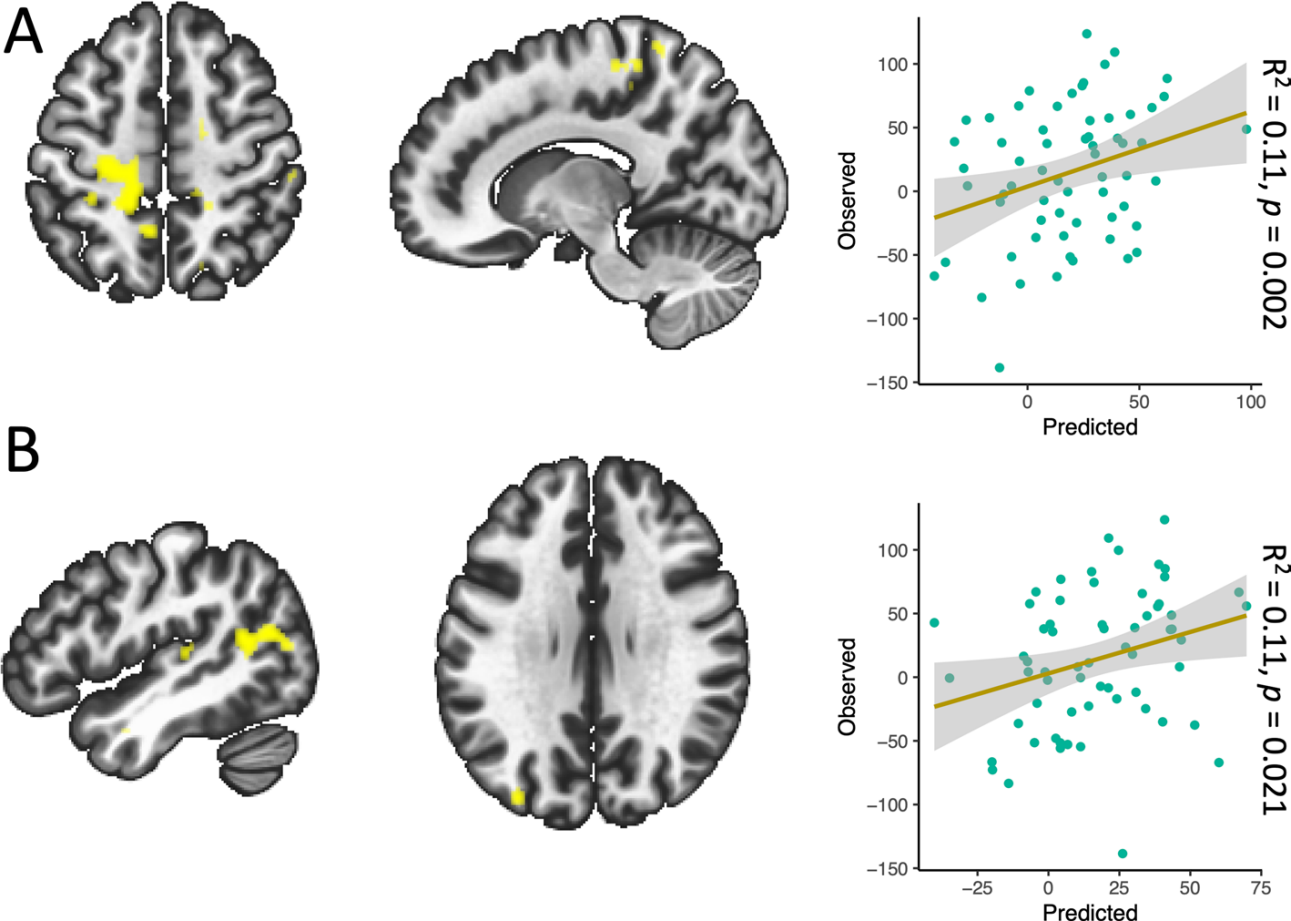
Summary figure of cluster regions predictive of change in time spent sedentary for each seed (**A** = aMCC, **B** = r-dA) and scatter plots of predicted vs observed values. Each summary figure represents the mean mask from each outer layer leave-one-out cross validation iteration that predicted the left-out subject’s change in time spent sedentary in the inner layer. For the aMCC seed (**A**), the mean cluster spanned regions in the primary motor cortex (axial slice view) and the supplementary motor area (sagittal slice view). For the r-dAI seed (**B**), the mean cluster mask spanned the middle temporal gyrus, angular gyrus and lateral occipital cortex.

In the dance and stretch and tone groups, baseline functional connectivity of the aMCC and the r-dAI was not predictive of change in time spent sedentary. In the walking group baseline functional connectivity between the aMCC and the M1/SMA predicted change in time spent sedentary (Table 2 and Fig. 3). Similarly, baseline functional connectivity between the r-dAI and the left temporoparietal/temporooccipital region (areas spanning the middle temporal gyrus, angular gyrus and lateral occipital cortex predicted change in time spent sedentary (Table 2 and Fig. 3). All results from these second level seed-based correlations were confirmed to hold in a whole-sample association analysis using conventional height-level statistical threshold of *P* < 0.001 and cluster threshold of *P* < 0.05 family wise error corrected (supplementary material 3).

## Discussion

The aims of the current study were to assess whether baseline functional connectivity of brain regions implicated in executive control and effort-based decision making could provide mechanistic predictions of change in time spent sedentary in older adults participating in a randomized control trial of exercise. In the walking group, participating in the most commonly found exercise intervention in the literature, we found that baseline behavioral measures were not predictive of change in time spent sedentary but functional connectivity of the aMCC and r-dAI were predictive. In the aerobic dance group and the control stretch and tone group, FC was not predictive of change in time spent sedentary, but baseline time spent sedentary was.

While our analysis of the objective measures of time spent sedentary did not reveal any differences between intervention conditions, previous research in this same sample^43^ demonstrated differences in out-of-session aerobic activity between intervention conditions, suggesting that the determinants of exercise and sedentary behaviors (in older adults participating in an exercise intervention) could differ between intervention types. Our result that FC predicted change in time spent sedentary in the walking group only is potentially in line with the idea that specific interventions may result in contextually different behaviors. Notwithstanding, many aerobic exercise interventions in older adults consist only of an active and a control intervention, making this conclusion hard to generalize. One possible interpretation of the differential predictiveness between our experimental conditions is perhaps related to statistical power. When we ran a down sampled analysis of the models using a randomly sampled N of 45 in the walking condition, the aMCC-to-M1/SMA relationship found in the wider walking sample was still present (supplementary material 4), but the r-dAI result was not. Interestingly, when running an exploratory analysis with all conditions combined (N = 143), the aMCC result disappears and the r-dAI result seen in the entire walking sample is present (supplementary material 5). This is perhaps suggestive of a lack of statistical power in the dance and stretch and tone samples, respectively, to detect a relationship between the FC of the r-dAI seed and change in time spent sedentary. Indeed, our power analysis in these intervention condition groups (supplementary material 6) found that we only have 64% power to detect an effect of the size seen in the walking condition. However, at the same time, these exploratory analyses lead to us speculate that the aMCC-to-M1/SMA FC result is perhaps specific to the walking condition. One possible explanation of the walking condition-specific result could lie in a previous analysis of this sample^43^ where participants in the walking group self-reported a reduction in the amount of out-of-session aerobic activity across the course of the 6-month intervention whereas those participating in the dance and stretch and tone conditions maintained their aerobic activity levels. In that prior analysis^43^, perceived intensity of the intervention sessions was associated with out-of-session aerobic activity, whereby higher perceptions of session activity were found for the walking group compared to the dance group and thus it was concluded that those in the walking group may have deemed the 3 times per week sessions as sufficient aerobic activity where as those in the dance and stretch and tone groups may have deemed their session to be necessary but insufficient, leading them to engage in more aerobic activity outside of the intervention sessions. Consequently, given the aMCC’ role in effort-based decision making, it is plausible that this mechanistic prediction of change in time spent sedentary is specific to those engaging in a walking intervention of a given intensity to be perceived as sufficient weekly aerobic exercise.

The main aim of this study was to ask whether resting state functional connectivity could provide mechanistic predictions of change in time spent sedentary. We chose our seed regions (aMCC and the r-dAI) as they have been consistently implicated in effort-based decision making and the integration of motor costs with reward outcomes^28,31,36,53^. Further, these same regions have been implicated in inhibitory control, which has been shown to be important to overcome the posited innate attraction towards effort minimization^26^. The function of the aMCC and its behavioral role has been highly debated (i.e. does it motivate effortful behaviors?^54,55^ or engage in decision-making and deployment of cognitive control?^56^). In an attempt to unify these theories, Holyrood and Yeung (2012) proposed that the aMCC supports the selection and maintenance of options and context-specific sequences of behavior directed towards particular goals. In line with this, it has been suggested that poorer monitoring of behavior by the aMCC (reflected as increased activity in the aMCC during error-related activity in a Go/NoGo task^57^) may increase the effort required to inhibit behaviors^38^. Highly relevant to our results, one previous study demonstrated that a network involving the aMCC and the SMA is critically involved in effort-based decision-making and the integration of motor costs into reward evaluation^28^. More importantly, the same study found that activity in the SMA was stronger in participants who tried to more activity avoid higher efforts^28^. It is plausible therefore that those participants in our study who increased their time spent sedentary were engaging in effort avoidance and/or poor behavioral monitoring, which is reflected as an increase in FC between the aMCC (involved in decision making where motor costs are evaluated) and the SMA (has higher activity during effort avoidance). Our aMCC seed result also extended into the primary motor cortex (M1) as well (Fig. 3A). While voluntary movements and internally-selected actions are more traditionally associated with the SMA^58^ and aMCC-to-SMA FC^54^, neural projections between the aMCC and M1 are present in primates^59,60^ and in fMRI studies, co-activation of the aMCC and motor regions have been seen in working memory tasks^61^. Activity in M1 has been found during mental effort and is likely involved in an attentional network linking behavioral responses to salient stimuli^62^. Indeed, the left medial portions of the cluster mapped onto the ventral attention network (VAN), a network involved in both attention^63^ and external awareness^64^. Further, activity in the left motor cortex has been shown to increase as the subjective value of effortful rewards increases^31^.

Higher FC between the r-dAI and a cluster overlapping the left temporoparietal and temporooccipital regions junction (regions covering the superior middle temporal gyrus and the inferior angular gyrus and lateral occipital gyrus) was also predictive of increases in time spent sedentary. The r-dAI has been proposed to provide an early cognitive control response^65^ and when mapping this result to a large functional network parcellation^66^, both the r-dAI and portions of this cluster (those in the temporoparietal junction (TPJ)) map onto a broad, bilateral VAN/salience network. Indeed, group level connectivity of the r-dAI ROI (Fig. 1D) shows positive FC with salience/VAN regions and is anticorrelated with the default mode network (a hallmark sign of the VAN). The VAN is said to be involved in re-direction of attention to behaviorally relevant stimuli^63,67^ and is implicated in more external awareness than the synonymous salience network^64^. Previous research using FC have shown the VAN to be predominantly (but not exclusively) lateralized to the right hemisphere^68^, nevertheless, bilateral TPJ was confirmed to be part of a broad VAN in a very large (N = 1000) study^66^. Additionally, the left TPJ’s inclusion in such a network seems to provide a distinct role beyond orientating attention to salient stimuli. For example, Webb and colleagues^64^ suggested that the left TPJ had a critical role in visual external awareness. The authors suggest that awareness can be disassociated from attention, and that significantly more attention may be drawn to a stimulus when subjects are aware of it^64^. Another study^69^ suggested that the left TPJ is functionally connected to other regions more associated with executive control and therefore may be more involved in the integration of contextual knowledge about salient stimuli. In accordance, the dAI has been suggested to be involved in awareness^70^. Further, the dAI and the aMCC are functionally connected at rest^71,72^ and across multiple tasks, the dAI and the aMCC are almost always coactivated^73^. Relevant to this study, the broad VAN network of brain regions that are implicated in our seed-based correlations have also been shown to change with advancing age^74^. Therefore, our results suggest that individual differences in the FC of this broad bilateral VAN, possibly engaging in external awareness, effort-based decision making and effort avoidance, in aging is predictive of changes in time spent sedentary in previously low-active older adults participating in a 6-month walking intervention.

Our results can only be interpreted in light of their limitations. The studies that have implicated the brain regions discussed have largely used task-based fMRI whereas we have relied upon intrinsic resting state FC. A future study to prospectively test the role of these brain regions in sedentary behaviors would provide stronger evidence of their mechanistic role. Furthermore, we restricted our analysis to a hypothesis-driven approach with a-prior defined seed regions. A future study may take a more data-driven approach surveying whole-brain functional connectivity in a more exploratory approach to assess the strength of as of yet unknown brain regions to predict change in time spent sedentary. The sample size in our study is relatively small and given the difficulty in objectively measuring sedentary behavior and the cost of running randomized control trials of exercise, we do not have an independent dataset on which to examine the generalizability of these results, nevertheless, cross-validation (which we employed) is one way to improve this generalizability. It is of note that the walking group contained participants randomized to either walking group or a walking group with a dietary supplement (see methods section for more details). No significant differences in behavioral and demographic variables were found between the groups and so we do not believe the dietary supplement will have affected our results. Regarding the accelerometry, because the Actigraph does not provide a reliable measure of body posture, we relied on a low-intensity proxy for sedentary behavior. As such we were unable to tease out very light activities and standing from overall sedentary time.

Here we show that individual differences in the baseline FC of multiple brain regions previously implicated in inhibitory control and effort-based decision making predict future change in sedentary time in low-active older adults participating in a 6-month walking intervention. Leveraging mechanistic predictors of future sedentary behaviors will potentially lead to targeted interventions that result in sustained behavioral change.

## Supplementary Information

Supplementary Information.

## Data Availability

All data will be provided upon reasonable request to the corresponding author, without reservation.

## Code availability

Code used in this manuscript includes R syntax for statistical analyses and will be shared upon request to the corresponding author.

## References

[1] Lee I-M (2012). Impact of physical inactivity on the world’s major non-communicable diseases. Lancet.

[2] Guthold R, Stevens GA, Riley LM, Bull FC (2018). Worldwide trends in insufficient physical activity from 2001 to 2016: a pooled analysis of 358 population-based surveys with 1·9 million participants. Lancet Glob. Health.

[3] Du Y (2019). Trends in adherence to the physical activity guidelines for americans for aerobic activity and time spent on sedentary behavior among US adults, 2007 to 2016. JAMA Netw. Open.

[4] Kohl HW (2012). The pandemic of physical inactivity: global action for public health. The Lancet.

[5] Ding D (2016). The economic burden of physical inactivity: a global analysis of major non-communicable diseases. The Lancet.

[6] Ding D (2017). The economic burden of physical inactivity: a systematic review and critical appraisal. Br. J. Sports Med..

[7] Kelly ME (2014). The impact of exercise on the cognitive functioning of healthy older adults: a systematic review and meta-analysis. Ageing Res. Rev..

[8] Northey JM, Cherbuin N, Pumpa KL, Smee DJ, Rattray B (2018). Exercise interventions for cognitive function in adults older than 50: a systematic review with meta-analysis. Br. J. Sports Med..

[9] Van Uffelen JGZ, Chin APMJM, Hopman-Rock M, Van Mechelen W (2008). The effects of exercise on cognition in older adults with and without cognitive decline: a systematic review. Clin. J. Sport Med..

[10] Gomes-Osman J (2018). Exercise for cognitive brain health in aging: a systematic review for an evaluation of dose. Neurol. Clin. Pract..

[11] Erickson KI (2019). Physical activity, cognition, and brain outcomes: a review of the 2018 physical activity guidelines. Med. Sci. Sports Exerc..

[12] Erickson KI (2011). Exercise training increases size of hippocampus and improves memory. Proc. Natl. Acad. Sci. U. S. A..

[13] Voss MW (2010). Plasticity of brain networks in a randomized intervention trial of exercise training in older adults. Front. Aging Neurosci..

[14] Buckner RL (2004). Memory and executive function in aging and AD: multiple factors that cause decline and reserve factors that compensate. Neuron.

[15] Luca CRD (2003). Normative data from the Cantab. I: development of executive function over the lifespan. J. Clin. Exp. Neuropsychol..

[16] Ng KK, Lo JC, Lim JKW, Chee MWL, Zhou J (2016). Reduced functional segregation between the default mode network and the executive control network in healthy older adults: a longitudinal study. Neuroimage.

[17] Nobis L (2019). Hippocampal volume across age: nomograms derived from over 19,700 people in UK Biobank. NeuroImage Clin..

[18] Spence JC, Rhodes RE, Carson V (2017). Challenging the dual-hinge approach to intervening on sedentary behavior. Am. J. Prev. Med..

[19] van der Ploeg HP, Hillsdon M (2017). Is sedentary behaviour just physical inactivity by another name?. Int. J. Behav. Nutr. Phys. Act..

[20] WHO. WHO |Physical Activity and Adults. *WHO* (2018).

[21] Conroy DE, Maher JP, Elavsky S, Hyde AL, Doerksen SE (2013). Sedentary behavior as a daily process regulated by habits and intentions. Health Psychol..

[22] Brand R, Cheval B (2019). Theories to explain exercise motivation and physical inactivity: ways of expanding our current theoretical perspective. Front. Psychol..

[23] Owen N (2011). Adults’ sedentary behavior: determinants and interventions. Am. J. Prev. Med..

[24] Szczuka Z, Banik A, Abraham C, Kulis E, Luszczynska A (2020). Associations between self-efficacy and sedentary behaviour: a meta-analysis. Psychol. Health.

[25] Falk EB (2015). Self-affirmation alters the brain’s response to health messages and subsequent behavior change. Proc. Natl. Acad. Sci..

[26] Cheval B (2020). Higher inhibitory control is required to escape the innate attraction to effort minimization. Psychol. Sport Exerc..

[27] Lachman ME, Lipsitz L, Lubben J, Castaneda-Sceppa C, Jette AM (2018). When adults don’t exercise: behavioral strategies to increase physical activity in sedentary middle-aged and older adults. Innov. Aging.

[28] Klein-Flügge MC, Kennerley SW, Friston K, Bestmann S (2016). Neural signatures of value comparison in human cingulate cortex during decisions requiring an effort-reward trade-off. J. Neurosci..

[29] Cheval B, Sarrazin P, Boisgontier MP, Radel R (2017). Temptations toward behaviors minimizing energetic costs (BMEC) automatically activate physical activity goals in successful exercisers. Psychol. Sport Exerc..

[30] Cheval B (2018). Behavioral and neural evidence of the rewarding value of exercise behaviors: a systematic review. Sports Med..

[31] Prévost C, Pessiglione M, Météreau E, Cléry-Melin M-L, Dreher J-C (2010). Separate valuation subsystems for delay and effort decision costs. J. Neurosci..

[32] Sockol MD, Raichlen DA, Pontzer H (2007). Chimpanzee locomotor energetics and the origin of human bipedalism. Proc. Natl. Acad. Sci..

[33] Ivanenko YP, Dominici N, Lacquaniti F (2007). Development of independent walking in toddlers. Exerc. Sport Sci. Rev..

[34] Selinger JC, O’Connor SM, Wong JD, Donelan JM (2015). Humans can continuously optimize energetic cost during walking. Curr. Biol..

[35] Vogt, B. A. Regions and Subregions of the Cingulate Cortex. 28 (2009).

[36] Porter BS, Li K, Hillman KL (2020). Regional activity in the rat anterior cingulate cortex and insula during persistence and quitting in a physical-effort task. eNeuro.

[37] Cai W, Ryali S, Chen T, Li C-SR, Menon V (2014). Dissociable roles of right inferior frontal cortex and anterior insula in inhibitory control: evidence from intrinsic and task-related functional parcellation, connectivity, and response profile analyses across multiple datasets. J. Neurosci..

[38] Garavan H, Hester R, Murphy K, Fassbender C, Kelly C (2006). Individual differences in the functional neuroanatomy of inhibitory control. Brain Res..

[39] Saghayi M (2020). Brain network topology predicts participant adherence to mental training programs. Netw. Neurosci..

[40] Whitfield-Gabrieli (2016). Brain connectomics predict response to treatment in social anxiety disorder. Mol. Psychiatry.

[41] Baniqued PL (2018). Brain network modularity predicts exercise-related executive function gains in older adults. Front. Aging Neurosci..

[42] Burzynska AZ (2020). Sensor-measured sedentariness and physical activity are differentially related to fluid and crystallized abilities in aging. Psychol. Aging.

[43] Ehlers DK, Fanning J, Awick EA, Kramer AF, McAuley E (2016). Contamination by an active control condition in a randomized exercise trial. PLOS ONE.

[44] Voss MW (2019). Nutritional supplementation boosts aerobic exercise effects on functional brain systems. J. Appl. Physiol. Bethesda Md.

[45] Troiano RP (2008). Physical activity in the United States measured by accelerometer. Med. Sci. Sports Exerc..

[46] Copeland JL, Esliger DW (2009). Accelerometer assessment of physical activity in active, healthy older adults. J. Aging Phys. Act..

[47] Whitfield-Gabrieli & Nieto-Castanon, A. Conn: a functional connectivity toolbox for correlated and anticorrelated brain networks. *Brain Connect.* **2**, 125–141 (2012). 10.1089/brain.2012.0073 22642651

[48] Van Dijk KRA (2009). Intrinsic functional connectivity as a tool for human connectomics: theory, properties, and optimization. J. Neurophysiol..

[49] Siegel JS (2017). Data quality influences observed links between functional connectivity and behavior. Cereb. Cortex N. Y. N.

[50] Behzadi Y, Restom K, Liau J, Liu TT (2007). A component based noise correction method (CompCor) for BOLD and perfusion based fMRI. Neuroimage.

[51] Wickham, H. *Welcome to the tidyverse*. (2019).

[52] Kuhn, M. *caret: Classification and Regression Training*. (2020).

[53] Bernacer J (2019). An amygdala-cingulate network underpins changes in effort-based decision making after a fitness program. NeuroImage.

[54] Mueller VA, Brass M, Waszak F, Prinz W (2007). The role of the preSMA and the rostral cingulate zone in internally selected actions. Neuroimage.

[55] Mulert C, Menzinger E, Leicht G, Pogarell O, Hegerl U (2005). Evidence for a close relationship between conscious effort and anterior cingulate cortex activity. Int. J. Psychophysiol..

[56] Kerns JG (2004). Anterior cingulate conflict monitoring and adjustments in control. Science.

[57] Hester R, Fassbender C, Garavan H (2004). Individual differences in error processing: a review and reanalysis of three event-related fMRI studies using the GO/NOGO task. Cereb. Cortex.

[58] Eccles JC (1982). The initiation of voluntary movements by the supplementary motor area. Arch. Für Psychiatr. Nervenkrankh..

[59] Morecraft RJ, Van Hoesen GW (1992). Cingulate input to the primary and supplementary motor cortices in the rhesus monkey: evidence for somatotopy in areas 24c and 23c. J. Comput. Neurol..

[60] Paus T (2001). Primate anterior cingulate cortex: where motor control, drive and cognition interface. Nat. Rev. Neurosci..

[61] Lenartowicz A, McIntosh AR (2005). The role of anterior cingulate cortex in working memory is shaped by functional connectivity. J. Cogn. Neurosci..

[62] Otto T, Zijlstra FRH, Goebel R (2018). Feeling the force: changes in a left-lateralized network of brain areas under simulated workday conditions are reflected in subjective mental effort investment. PLOS ONE.

[63] Corbetta M, Patel G, Shulman GL (2008). The reorienting system of the human brain: from environment to theory of mind. Neuron.

[64] Webb TW, Igelström KM, Schurger A, Graziano MSA (2016). Cortical networks involved in visual awareness independent of visual attention. Proc. Natl. Acad. Sci..

[65] Ham T, Leff A, de Boissezon X, Joffe A, Sharp DJ (2013). Cognitive control and the salience network: an investigation of error processing and effective connectivity. J. Neurosci..

[66] Yeo BTT (2011). The organization of the human cerebral cortex estimated by intrinsic functional connectivity. J. Neurophysiol..

[67] Corbetta M, Shulman GL (2002). Control of goal-directed and stimulus-driven attention in the brain. Nat. Rev. Neurosci..

[68] Fox MD, Corbetta M, Snyder AZ, Vincent JL, Raichle ME (2006). Spontaneous neuronal activity distinguishes human dorsal and ventral attention systems. Proc. Natl. Acad. Sci..

[69] Kucyi A, Hodaie M, Davis KD (2012). Lateralization in intrinsic functional connectivity of the temporoparietal junction with salience- and attention-related brain networks. J. Neurophysiol..

[70] Craig AD (2011). Significance of the insula for the evolution of human awareness of feelings from the body. Ann. N. Y. Acad. Sci..

[71] Medford N, Critchley HD (2010). Conjoint activity of anterior insular and anterior cingulate cortex: awareness and response. Brain Struct. Funct..

[72] Taylor KS, Seminowicz DA, Davis KD (2009). Two systems of resting state connectivity between the insula and cingulate cortex. Hum. Brain Mapp..

[73] Craig AD (2009). How do you feel—now? The anterior insula and human awareness. Nat. Rev. Neurosci..

[74] Deslauriers J, Ansado J, Marrelec G, Provost J-S, Joanette Y (2017). Increase of posterior connectivity in aging within the ventral attention network: a functional connectivity analysis using independent component analysis. Brain Res..

