# Supplementary material for: Resting state functional connectivity provides mechanistic predictions of future changes in sedentary behavior
Source: Sci Rep. 2022 Jan 18;12:940. doi: 10.1038/s41598-021-04738-y (PMC8766514; doi:10.1038/s41598-021-04738-y)
Supplement: Supplementary file 1 — Supplementary Information. [file 41598_2021_4738_MOESM1_ESM.docx]

**Supplementary material**

**Resting State Functional Connectivity Provides Mechanistic Predictions of Future Changes in Sedentary Behavior**

Timothy P. Morris^a^, Aaron Kucyi^a^, Sheeba Arnold Anteraper^a^, Maiya Rachel Geddes^b,c^, Alfonso Nieto-Castañon^a^, Agnieszka Burzynska^d^, Neha P. Gothe^e,f^, Jason Fanning^g^, Elizabeth A. Salerno^h^, Susan Whitfield-Gabrieli^a,i^, Charles H. Hillman^a,j^, Edward McAuley^e,k^, Arthur F. Kramer^a,e^

^a^ Department of Psychology, Northeastern University, Boston, MA, United States

^b^ Department of Neurology and Neurosurgery, Montreal Neurological Institute, McGill University, Canada

^c^ Brigham and Women’s Hospital, Harvard Medical School, United States

^d^ Department of Human Development and Family Studies, Colorado State University, USA

^e^ Beckman Institute for Advanced Science and Technology, University of Illinois at Urbana Champaign, United States

^f^ Department of Kinesiology and Community Health, University of Illinois at Urbana-Champaign, Urbana, IL, United States

^g^ Department of Health and Exercise Sciences, Wake Forrest University, North Carolina, USA

^h^ Division of Public Health Sciences, Department of Surgery, Washington University School of Medicine in St. Louis, St. Louis, MO

^i^ McGovern Institute for Brain Research, Department of Brain and Cognitive Sciences, Massachusetts Institute of Technology, Cambridge, MA, United States.

^j^ Department of Physical Therapy, Movement, and Rehabilitation Sciences, Northeastern University, Boston, MA, United States

^k^ Department of Kinesiology and Community Health, University of Illinois at Urbana-Champaign, Urbana, IL, United States

1. Testing for differences between walking groups

| Supplementary table 1: Testing for differences between walking groups | | | |
| --- | --- | --- | --- |
|  | Walk | Walk + | p |
| n | 29 | 34 |  |
| age (mean (SD)) | 65.03 (4.16) | 65.56 (4.94) | 0.653 |
| Pre sedentary time (mean (SD)) | 540.09 (104.10) | 532.04 (80.44) | 0.731 |
| Post sedentary time (mean (SD)) | 543.36 (102.78) | 555.58 (95.63) | 0.627 |
| gender = 2 (%) | 21 (72.4) | 24 (70.6) | 1.000 |

1. MRI quality control

The following plots illustrate quality assurance measures of the resting state fMRI data included in this analysis for 145 subjects. Note, 2 were removed from the analyses for having greater than 30 invalid scans flagged.


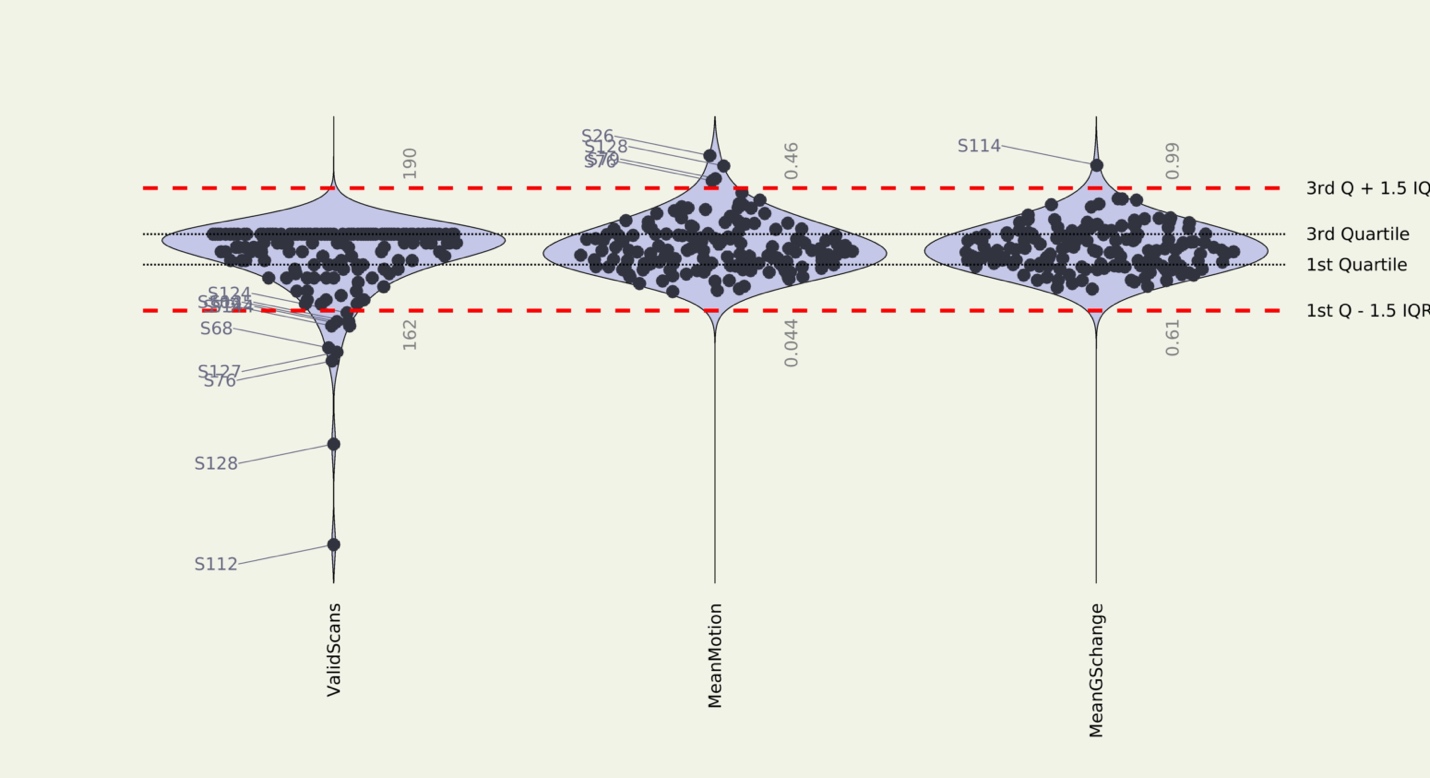


**Supplementary figure 1.** Quality assurance plot, motion parameters.


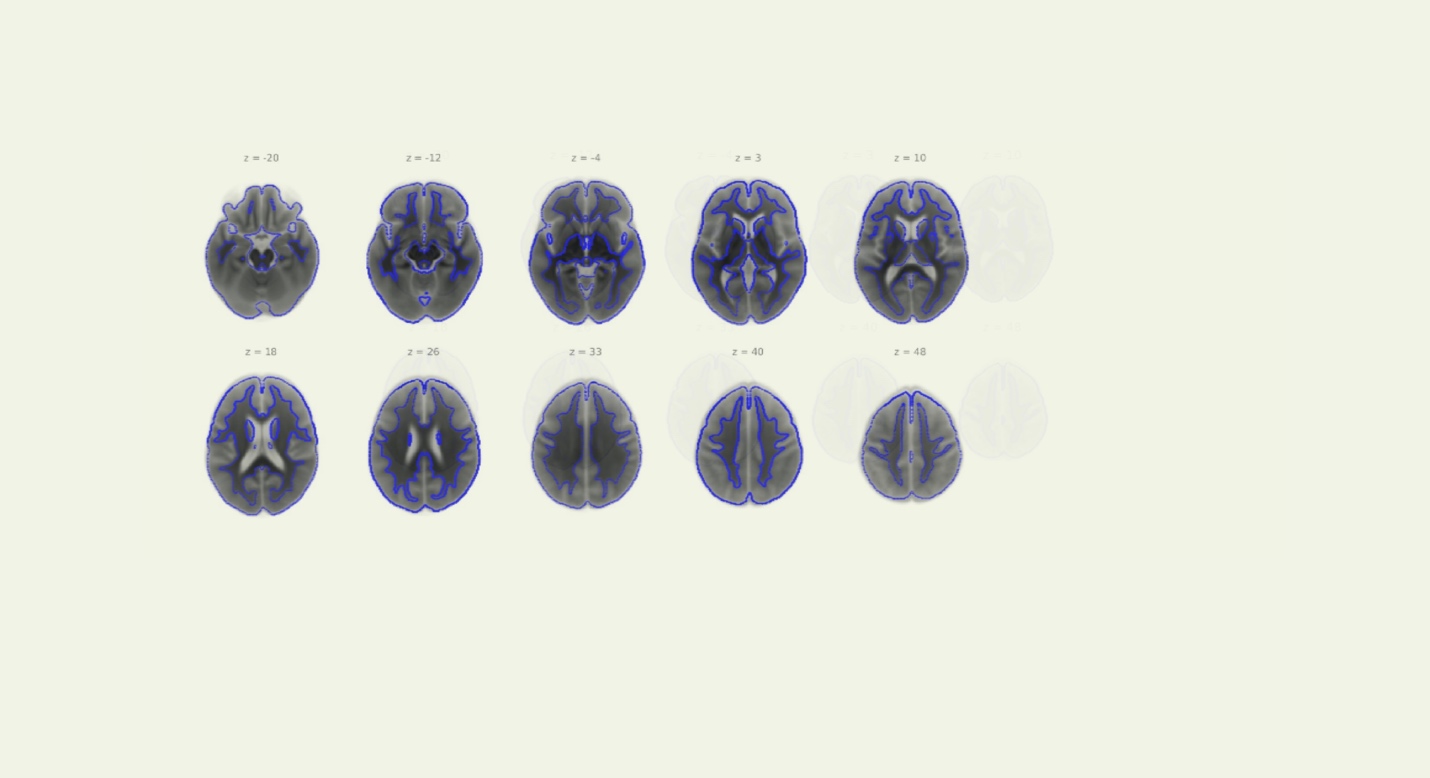


**Supplementary figure 2.** Quality assurance plot, structural normalization.


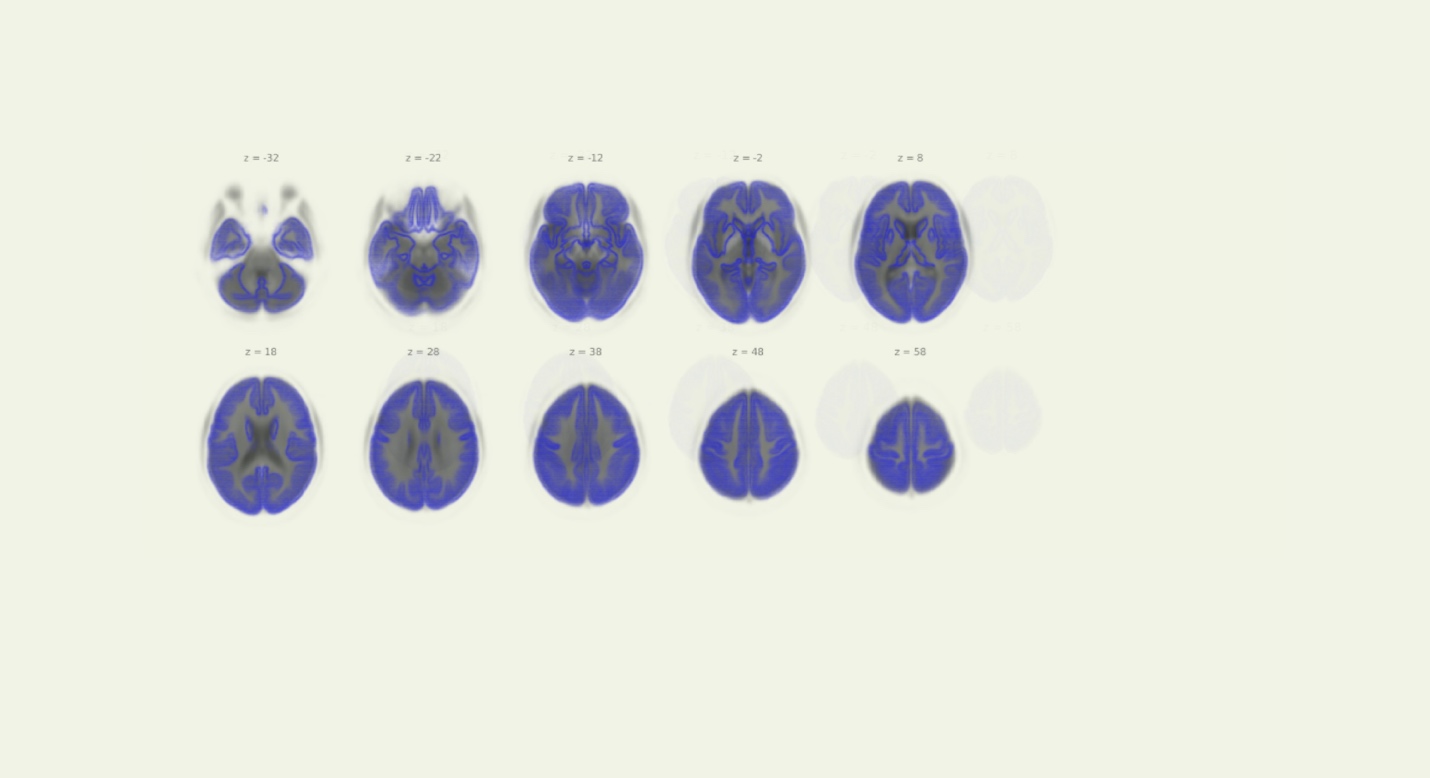


**Supplementary figure 3.** Quality assurance plot, functional data with structural overlay


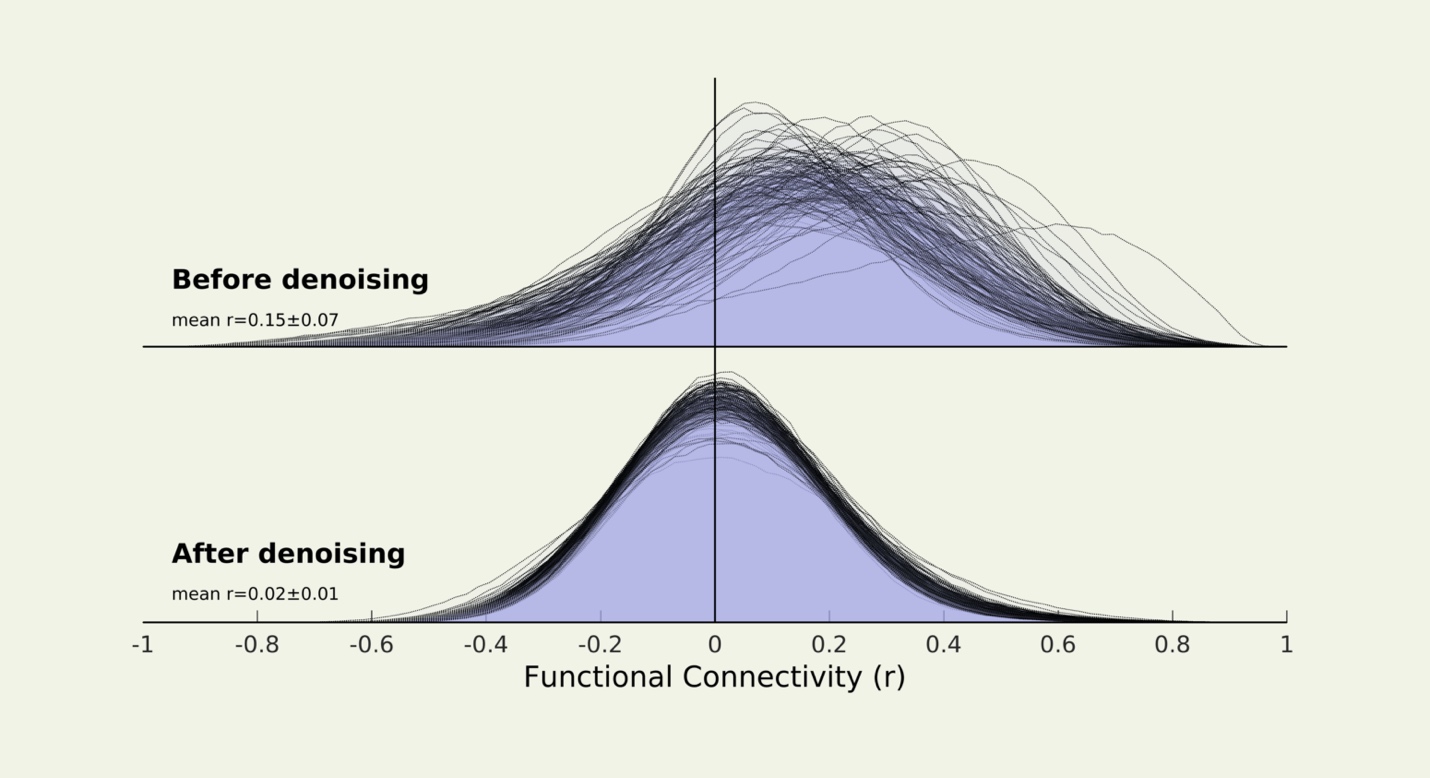


**Supplementary figure 4.** Quality assurance plot, distribution of functional connectivity data before and after denoising

1. Confirmatory seed-based correlation analysis with conventional voxel and cluster thresholding

In a confirmatory step to ensure that our results conformed to conventional thresholding for multiple comparisons, we estimated our second-level seed-based analyses using a height threshold (voxel level *p*<0.001) and a family-wise corrected cluster-extent threshold (*p* FWE<0.05).

In the walking group the aMCC seed revealed a significant positive cluster (voxel *p*<0.001 and FWE cluster-level *p*<0.05 correction) spanning the motor and the supplementary motor areas (peak MNI: -24 -20 +52, size = 425 voxels) associated with change in sedentary time (*T*(58) = 3.47, *p*_fwe_ = <0.001). In the same walking group, functional connectivity between the r-dAI and a significant (voxel *p*<0.001 and FWE cluster-level *p*<0.05 correction) cluster in the left temporoparietal/temporooccipital region (areas spanning the middle temporal gyrus, angular gyrus and lateral occipital cortex (peak MNI: -50 -56 +12, size = 194 voxels)) was also associated with change in sedentary time (*T*(58) = 3.47, *p*_fwe_ = <0.001).

1. Multiple comparison corrections

We controlled for multiple comparisons across all models (2 seeds and 1 behavior models for each of the 3 groups). For those FC models that did not find an association using seed-based correlations (and thus no Pvalue was provided) we used a p value of 1. All relationships that were significant in the uncorrected models remained significant after multiple comparison corrections with false discovery rate.

| **Model** | **pvalue** | **fdr pvalue** |
| --- | --- | --- |
| **Walking FC 1** | 0.002 | 0.009 |
| **Walking FC 2** | 0.021 | 0.047 |
| **Stretch behavior** | 0.001 | 0.009 |
| **Dance behavior** | 0.003 | 0.009 |
| **Walking behavior** | 0.22 | 0.396 |
| **Dance FC 1** | 1 | 1 |
| **Dance FC 2** | 1 | 1 |
| **Stretch FC 1** | 1 | 1 |
| **Stretch FC 2** | 1 | 1 |

1. Replication of the seed-based correlation between the FC of the aMCC and a cluster spanning M1 and the SMA in a randomly-selected down sampled group of 45 participants in the walking group


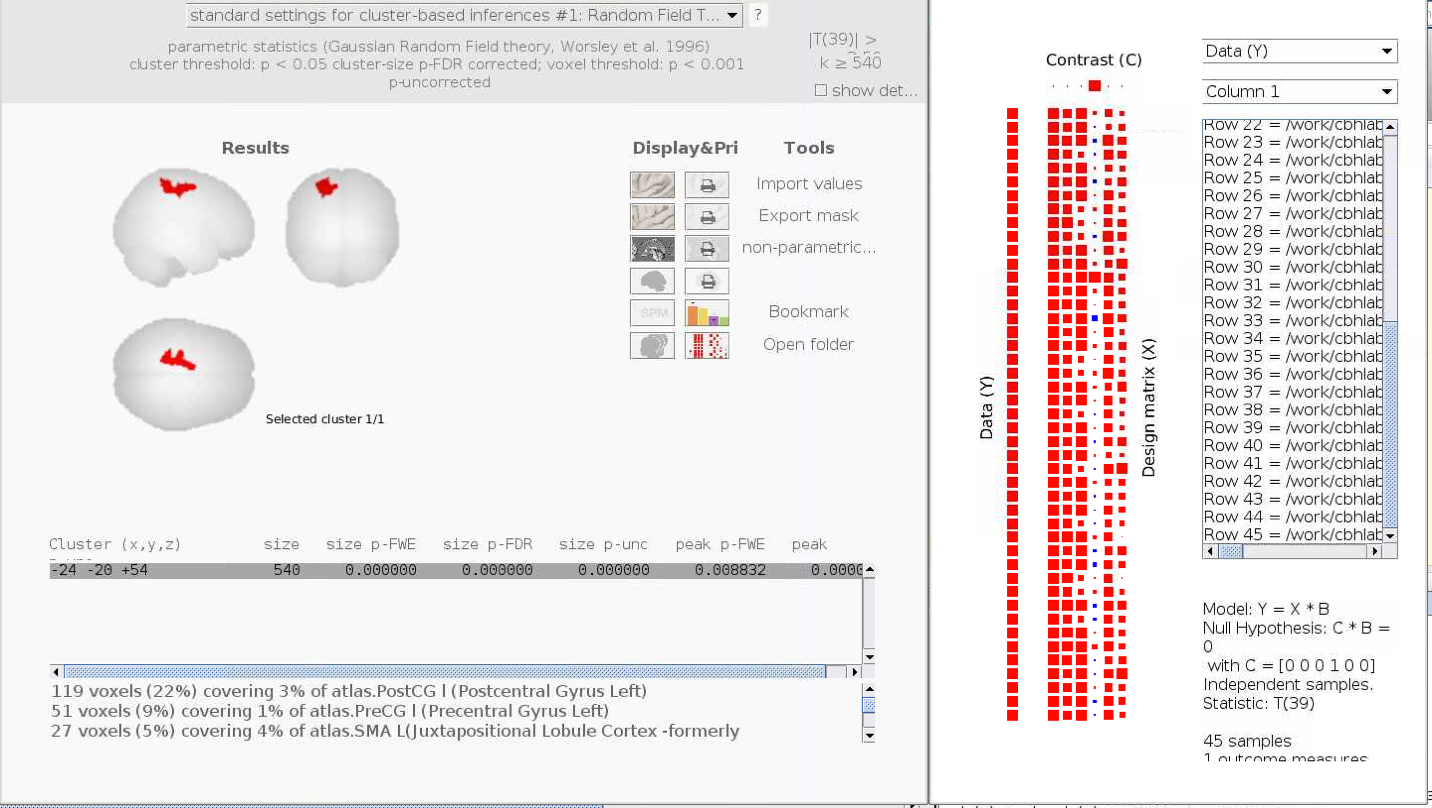


**Supplementary figure 6.** Result of the seed-based correlation between the aMCC and a cluster spanning M1 and the SMA in a down sampled N of 45 in the walking condition.

1. Replication of the seed-based correlation between the FC of the r-dAI and a cluster in the right temporoparietal/temporooccipital junction in a combined sample of participants across all intervention condition (N=143) controlling for all previous covariates and group assignment.


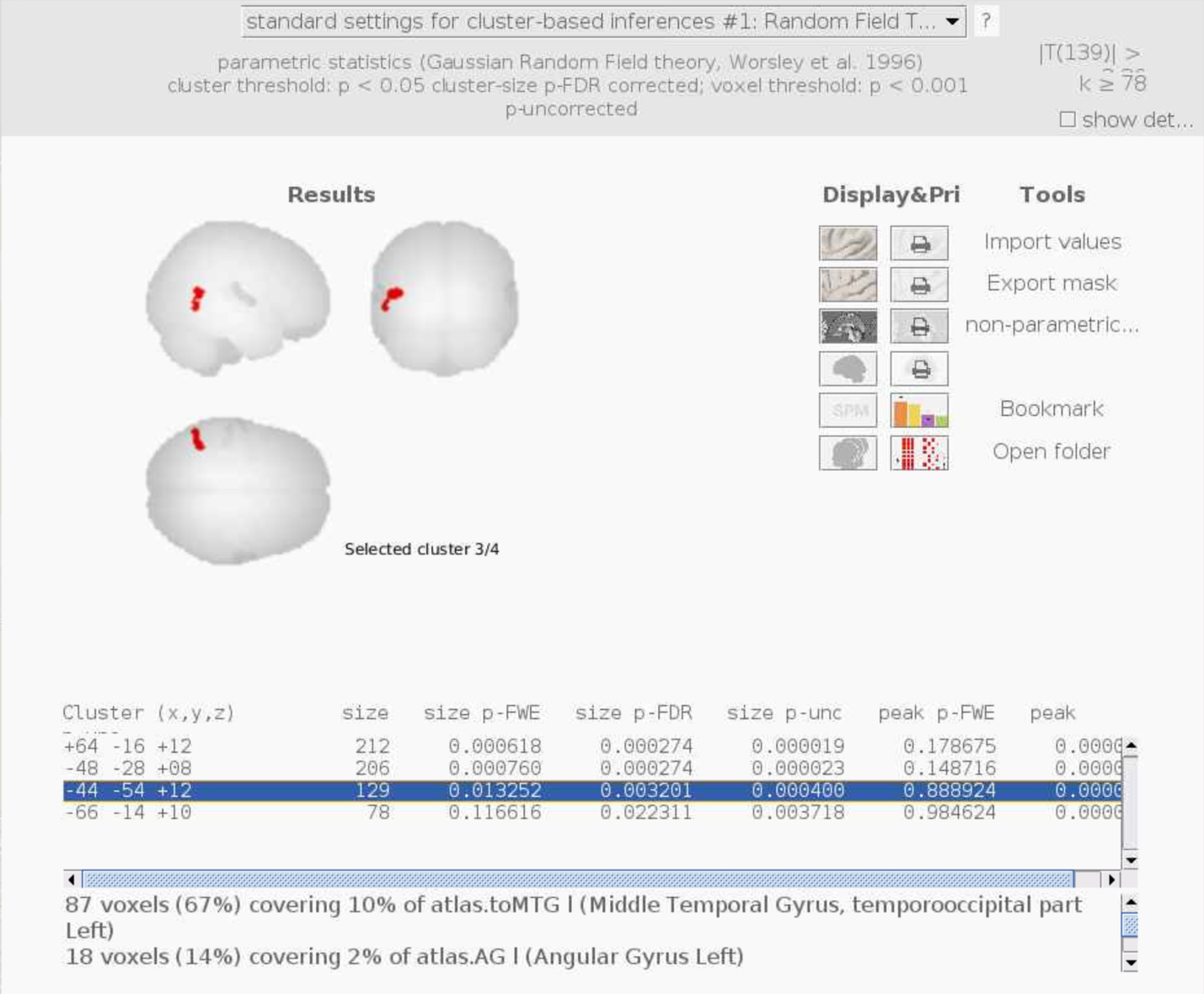


**Supplementary figure 7.** Result of the seed-based correlation between the r-dAI and a cluster within the temporooccipital junction in the entire sample (N=143). (*T*(136) = 3.36, *p*_fwe_ = <0.001) peak MNI: -44 -54 +12.

1. Power analysis

We performed a power analysis (in R using the “pwr” package) on our sample size to ensure sufficient power was gained to detect a true effect. Based on our sample size for the walking condition (N = 63) and an assumed type I error rate of 0.05, we calculated an estimated 88% power to detect an effect size of 0.21 from a general linear model with 4 covariates. For the dance and stretch and tone condition sample sizes (N=40), only 64% power was detected for the same effect size.
